# ProBDNF and its receptors in immune-mediated inflammatory diseases: novel insights into the regulation of metabolism and mitochondria

**DOI:** 10.3389/fimmu.2023.1155333

**Published:** 2023-04-18

**Authors:** Qiao Li, Yue-Zi Hu, Shan Gao, Peng-Fei Wang, Zhao-Lan Hu, Ru-Ping Dai

**Affiliations:** ^1^ Department of Anesthesiology, The Second Xiangya Hospital, Central South University, Changsha, Hunan, China; ^2^ Anesthesia Medical Research Center, Central South University, Changsha, Hunan, China; ^3^ Clinical Laboratory, The Second Hospital of Hunan University of Chinese Medicine, Changsha, Hunan, China

**Keywords:** ProBDNF, p75^NTR^, sortilin, immune-mediated inflammatory diseases, mitochondria, metabolism

## Abstract

Immune-mediated inflammatory diseases (IMIDs) consist of a common and clinically diverse group of diseases. Despite remarkable progress in the past two decades, no remission is observed in a large number of patients, and no effective treatments have been developed to prevent organ and tissue damage. Brain-derived neurotrophic factor precursor (proBDNF) and receptors, such as p75 neurotrophin receptor (p75^NTR^) and sortilin, have been proposed to mediate intracellular metabolism and mitochondrial function to regulate the progression of several IMIDs. Here, the regulatory role of proBDNF and its receptors in seven typical IMIDs, including multiple sclerosis, rheumatoid arthritis, systemic lupus erythematosus, allergic asthma, type I diabetes, vasculitis, and inflammatory bowel diseases, was investigated.

## 1 Introduction

Immune-mediated inflammatory diseases (IMIDs) are a group of highly disabling chronic diseases characterized by immune dysregulation and chronic inflammation as the basic manifestation, which affect different organs and systems (1, 2). The prevalence of IMIDs in well-developed countries is about 5%–8% (1), and its global incidence gradually increases (3, 4). IMIDs consist of more than 100 different types of diseases, such as multiple sclerosis (MS), rheumatoid arthritis (RA), systemic lupus erythematosus (SLE), allergic asthma, type I diabetes (T1D), vasculitis, and inflammatory bowel diseases (IBD), involving multiple disciplinary fields (5–8). At present, no effective targeted treatment has been developed for IMIDs; thus, exploring novel therapeutic targets for IMIDs is necessary.

Based on previous reports, targeting brain-derived neurotrophic factor (BDNF), BDNF precursor (proBDNF), and its receptors exert therapeutic effects in IMIDs. The administration of anti-proBDNF monoclonal antibodies (mAb-proB) can effectively improve the neurological score and reduce the number of lymphocytes in the experimental autoimmune encephalomyelitis (EAE) mouse model, a classic model of MS. ProBDNF and its high-affinity receptor, p75 neurotrophin receptor (p75^NTR^), were upregulated in peripheral blood mononuclear cells (PBMCs) from patients with RA compared with healthy controls. Treatment of the extracellular domain of p75^NTR^ (p75^ECD^) can significantly relieve inflammatory pain in collagen-induced arthritis (CIA) model mice, which is a standard RA model. MAb-proB could also reduce the production of auto-antibodies and attenuate kidney injury in SLE by altering the mitochondrial respiratory chain complex transcription level and cholesterol metabolism (9, 10).

ProBDNF plays an important role in the mitochondria-mediated release of cytochrome C (cyt C) to regulate cell death by binding the receptor complex of p75^NTR^ and sortilin (11). P75^NTR^ signaling could regulate glucose uptake (12), and sortilin is a crucial regulatory molecule of lipid metabolism (13, 14). In this review, the potential effects of proBDNF and its receptors on glucose, lipid, and mitochondrial metabolism in IMIDs were explored and concluded.

## 2 ProBDNF and its receptors

### 2.1 Role of proBDNF signaling in immune cells and IMIDs

**Figure 1:**
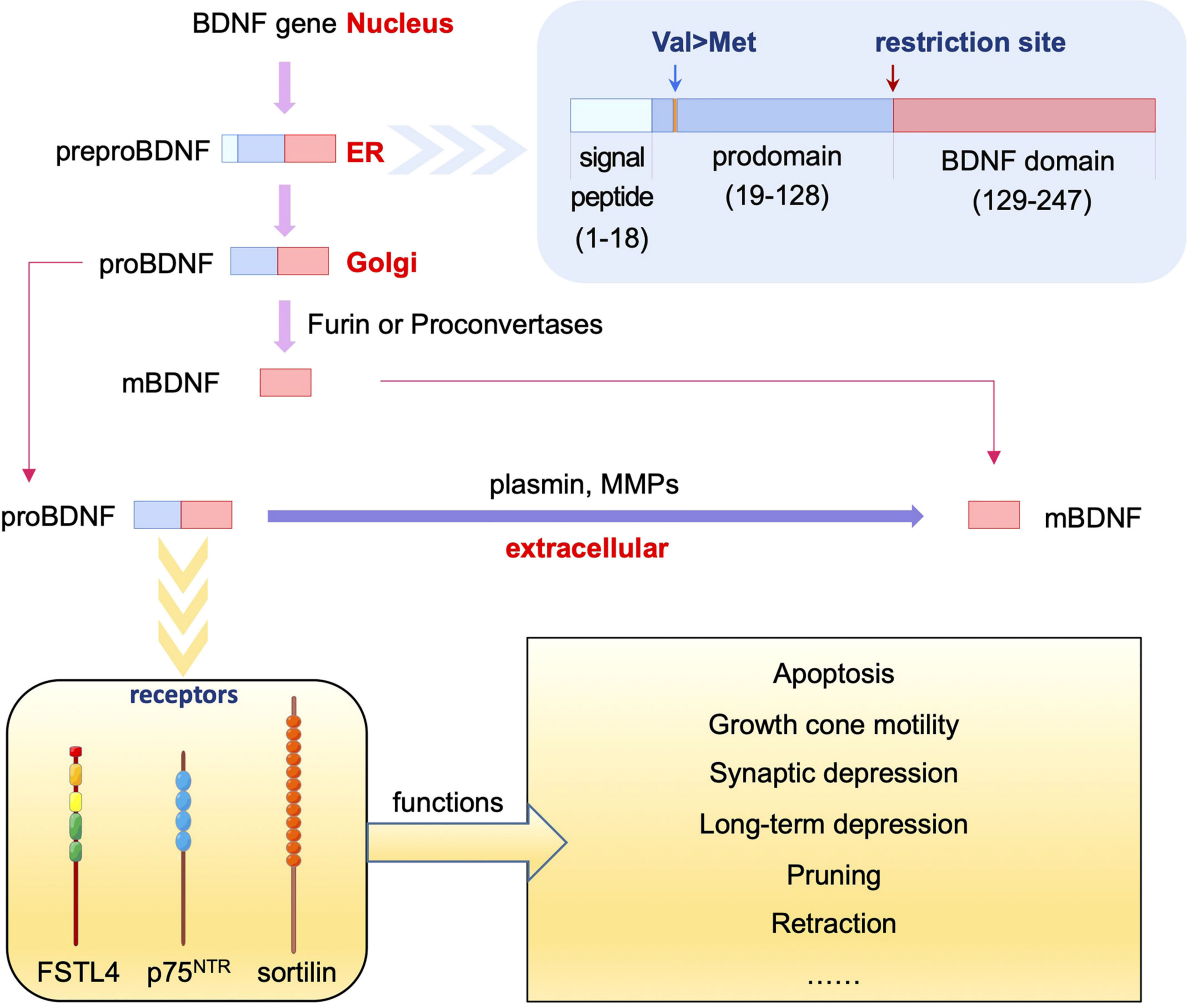
Processing and function of proBDNF and its receptors. The BDNF gene produces preproBDNF protein in the ER, which is processed to proBDNF in Golgi. ProBDNF may be cleaved into mBDNF intracellularly by furin or proconvertases, or extracellularly by plasmin or MMPs. ProBDNF binds to p75^NTR^, sortilin or FSTL4 to exert different effects including apoptosis, growth cone motility, synaptic depression, long-term depression, pruning, retraction and other functions.

BDNF is a widely studied member of the neurotrophin family. The BDNF gene produces preproBDNF protein in the endoplasmic reticulum, which is processed to proBDNF in the Golgi for sorting into either constitutive or regulated secretory vesicles (15). ProBDNF may be cleaved into mature BDNF (mBDNF) intracellularly by furin in the trans-Golgi network or proconvertases in secretory vesicles. ProBDNF and mBDNF can be secreted from neurons in an activity-dependent manner. In addition, proBDNF can be cleaved into mBDNF extracellularly by plasmin or selective matrix metalloproteinases (MMPs), including MMP3, MMP7, and MMP9 (16, 17) **(** **Figure 1** **)**. ProBDNF and mBDNF play contrasting biological roles in the synaptic structure, plasticity, transmission, and activity in the central nervous system (CNS) (18). MBDNF binds tyrosine kinase receptor B (TrkB) to promote cell survival, whereas proBDNF induces apoptosis (16, 19–22).

ProBDNF consists of 129 amino acids in the N-terminal prodomain and 118 amino acids in the C-terminal mature domain (23). In the CNS, proBDNF is primarily located in the spinal cord dorsal horn, nuclei tractus solitarius, spinal trigeminal nuclei, spinal trigeminal nuclei, hypothalamus, and amygdala (24). Multiple expressions of proBDNF are also found in peripheral tissues, such as the skin, intestine, adrenal gland, pituitary gland, spinal cord dorsal horn (24), and liver (25). ProBDNF inhibits neural stem cell proliferation, differentiation, and migration, and reduces the number of neurons, oligodendrocytes, and astrocytes, whereas anti-proBDNF antibodies reverse neural stem cell proliferation and differentiation (26). Recombinant proBDNF protein modulates neuronal architecture and alters the long-term plasticity in the hippocampus *in vitro*; however, the role of endogenous proBDNF remains unclear (27).

ProBDNF can be expressed not only in the CNS and peripheral tissues, but also in immune cells such as monocytes/macrophages (28), T cells (29, 30), and B cells (9). In addition, proBDNF plays an important role in IMIDs. Studies have shown that peripheral macrophages can secrete proBDNF under certain inflammatory conditions (31, 32). Our previous studies also indicated that proBDNF, as a key mediator of neuroinflammation in spinal cord injury and inflammatory pain, is released from infiltrating macrophages, and its activity can be reduced using mAb-proB (31, 32). The increased proBDNF expression on monocytes/macrophages promotes inflammatory response in type A aortic dissection patients with severe systemic inflammation (28). The injection of lipopolysaccharide (LPS) could induce the upregulation of proBDNF in CD4^+^ T cells (30, 33) and further modulate sepsis-associated encephalopathy (30). The activation of Rac1 and TRPM7 channels in innate immune cells, such as microglia, can mediate the combination of proBDNF and p75^NTR^, which induced a sustained increase in intracellular Ca^2+^ concentration and enhanced IFN-γ-induced nitric oxide production (34). Interestingly, proBDNF does not always exert pro-inflammatory effects. Our recent study revealed that endogenous proBDNF in proinflammatory monocytes/macrophages played a protective role by regulating MMP‐9 signaling in acute myocardial infarction (AMI). Administration of mAb-proB skewed monocytes/macrophages into a proinflammatory phenotype after AMI (35).

Studies have shown that the receptors binding to proBDNF include p75^NTR^ (9), sortilin (32, 36), and follistatin Like 4 (FSTL4) (37), which play different roles in nerve-immunity-endocrine network **(** **Figure 1** **)**.

P75^NTR^, a member of the tumor necrosis factor receptor superfamily, is a receptor with high affinity to proBDNF. ProBDNF binds to p75^NTR^ to promote cell death and inhibit long-term potentiation and neuronal axon outgrowth (38, 39), and to be involved in regulating neurotransmitter release in the entorhinal cortex (18). ProBDNF does not always induce cell death but can also be involved in regulating synaptic activity, pruning, and network reorganization (18). In the CNS, proBDNF/p75^NTR^ weakens synaptic transmission under the synergistic effect of sortilin protein, negatively regulating synaptic plasticity; triggering neuronal apoptosis, axon pruning, and axon collapse; and exerting biological effects contrary to mBDNF (27, 39–43).

By the end of the 1980s, studies have found that in addition to neurons, the expression of p75^NTR^ can also be detected on various immune cells such as PBMCs (44). Increased expression of proBDNF and p75^NTR^ could be detected in PBMCs after strenuous exercise in normal adult males (45). This result indicates that when vigorous exercise reaches a certain threshold, immune cells produce proBDNF in an autocrine and/or paracrine manner to regulate apoptotic pathways. ProBDNF binds to p75^NTR^ on monocytes, thereby activating the NF-κB pathway and enhancing the immune function of peripheral lymphocytes (29, 46). In addition, B cells can self-secrete proBDNF, and the combination of proBDNF and p75^NTR^ with the cooperation of sortilin transporter induces apoptosis of B cells (47).

Sortilin belongs to the VPS10 family (48) and exerts a dual function involved in intracellular protein transport and cell signal transduction to regulate neuronal death or survival and the process of immune cells. It is primarily expressed on macrophages and dendritic cells and, to a lesser extent, on B and T cells (49). In neurons, sortilin serves as a membrane-bound coreceptor complex with p75^NTR^ to facilitate the affinity of proBDNF-binding p75^NTR^ to signal cell death (40, 50, 51). Studies have pointed out that sortilin plays a role in the survival and activation of B cells by regulating the transport of BDNF, and silencing sortilin can decrease the secretion of BDNF and increase the apoptosis of B cells (47). The loss of sortilin on cytotoxic T cells can decrease the release of IFN-γ and increase the expression of granzyme A in T cells (52). Rogers et al. found that sortilin binds to p75^NTR^ to mediate NK cell apoptosis, and blocking sortilin with neurotensin could reduce NK cell death (53). In addition, sortilin plays a role in the antigen processing of DCs (49).

FSTL4, also known as SPARC-related protein containing immunoglobulin domains 1 (SPIG1), belongs to the SPARC family (54). It consists of a signal peptide, a follistatin-like domain, an extracellular calcium-binding domain with two EF-hand motifs, and two immunoglobulin-like domains (54, 55). Based on previous reports, FSTL4 negatively regulates BDNF maturation by binding to proBDNF (54). Furthermore, Suzuki et al. confirmed that the extinction of aversive memories was enhanced in *Spig1*-KO mice, revealing that FSTL4 suppresses synaptic plasticity in the extinction of inhibitory avoidance memory, which might be associated with its negative regulation on BDNF maturation from proBDNF (55). Our study indicated that the proBDNF/FSTL4 pathway contributed to neuronal apoptosis, whereas its downstream signaling remained unknown (37).

### 2.2 ProBDNF and its receptors mediate the regulation of metabolism and mitochondria

ProBDNF treatment could cause a significant dose-related decrease in mitochondria membrane potential, but it could not alter LDH released from dying or damaged cortical neurons (56). A study reported that proBDNF binds to p75^NTR^ and sortilin to induce mitochondrial apoptosis by inhibiting the PI3K signaling pathway, which contributes to neuronal apoptosis in dorsal root ganglia (57). The age-dependent increase in proBDNF expression was found to be associated with a decrease in mitochondrial metabolism activity and content of epididymal white adipose tissue (eWAT) (58). Therefore, upregulated proBDNF expression in adipose progenitor cells of aged animals triggered the death of adipocytes, leading to the infiltration of immune cells and disruption of metabolic fitness. In addition, val66Met single-nucleotide polymorphism in the prodomain of BDNF induces altered trafficking of BDNF within neurons and decreases the activity-dependent secretion of mBDNF (59). Moreover, this variant could increase food intake in mice, which is consistent with the orexigenic activity of p75^NTR^ (60). Based on these reports, proBDNF may exert stronger control of energy regulation. ProBDNF could be cleaved to BDNF if energy supply is sufficient even excessive, converting the orexigenic activity to anorexigenic.

**Figure 2:**
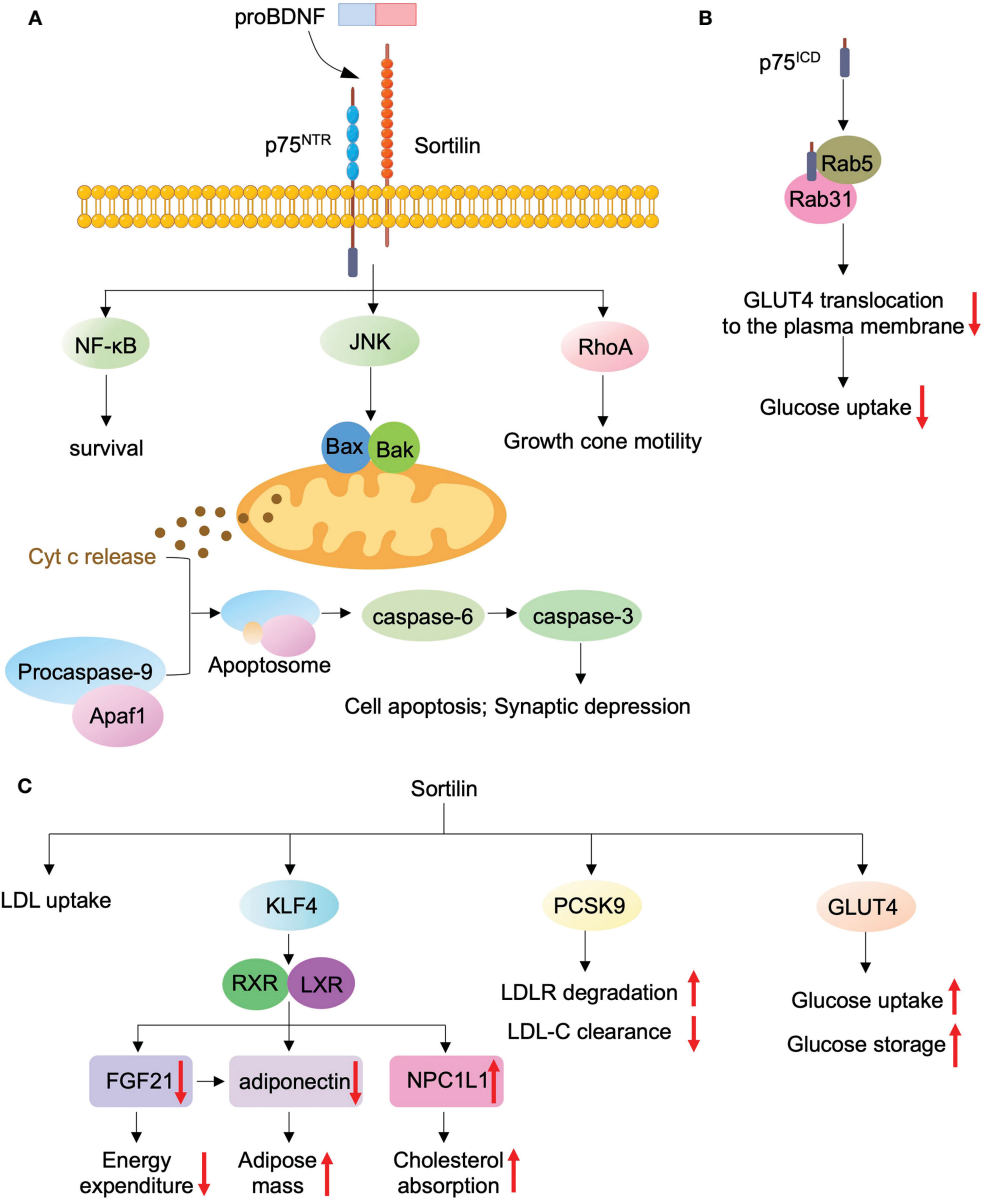
ProBDNF and their receptors mediate the regulation of metabolism and mitochondria. ProBDNF binds specifically to p75^NTR^ through interaction with sortilin, which induces JNK signaling to increase the release of cytochrome C from mitochondria and promote cell apoptosis and synaptic depression. ProBDNF binds p75^NTR^ and sortilin promotes growth cone motility and cell survival *via* activating RhoA and NF-κB, respectively **(A)**. P75^ICD^ forms a complex with Rab5 and Rab31 GTPases to enhance glucose uptake *via* promoting GLUT4 translocation to the plasma membrane **(B)**. Sortilin also enhances LDL uptake and LDLR degradation, whereas decreases clearance of LDL-C. Sortilin promotes glucose uptake and storage *via* interacting with GLUT4 **(C)**.

Three main downstream signaling pathways are identified after p75^NTR^ activation, including RhoA, JNK, and NF-κB pathways (43, 46, 61, 62). Among these, the canonical pathway mediating cell death is JNK signaling activated by the p75^NTR^/sortilin complex, which causes the activation of proapoptotic Bak and Bax proteins in the Bcl-2 family, cyt C release from the mitochondria, and formation of apoptosome, followed by caspase9/3 activation, which contributes to cell death and synaptic depression (11, 63) **(** **Figure 2A** **)**. Although p75^NTR^ signaling has been well-characterized over the years, few studies have well interpreted the signaling pathways involved to exert effects on mitochondrial metabolism. Furthermore, signaling cascades required for the regulation of mitochondrial metabolism remain unclear.

P75^NTR^ is expressed in WAT, skeletal muscle, and liver, and it serves as a central regulator of glucose metabolism and energy balance (64, 65). P75^NTR^ knockout (p75^NTR−/−^) mice showed improved glucose tolerance, insulin sensitivity, and inhibited hepatic glucose production (64). In addition, p75^NTR−/−^ mice are protected from high-fat diet (HFD)-induced obesity as a result of enhanced energy expenditure and insulin sensitivity (65). In adipocytes, the intracellular domain of p75^NTR^ (p75^ICD^) forms a complex with Rab5 and Rab31 GTPases to regulate Glut4 trafficking **(** **Figure 2B** **)** (64). P75^NTR^ also directly interacts with protein kinase A (PKA) and mediates cAMP signaling in adipocytes, thereby inhibiting lipolysis, and thermogenesis. Adipocyte-specific p75^NTR^ knockout or transplantation of WAT from p75^NTR−/−^ into wild-type mice fed a HFD protects mice against weight gain and insulin resistance; thus, the p75^NTR^/PKA signaling pathway is a potential therapeutic target for metabolic disorders (65). However, p75^NTR^ serves as a neurotrophin receptor; thus, future studies must be conducted to determine whether or not p75^NTR^ regulates PKA signaling in the CNS and the integral mechanism of p75^NTR^-regulated-metabolism affecting metabolic diseases.

*Sort1* (encoding sortilin), a novel lipid gene, could regulate body weight and cholesterol metabolism (66). This gene also has a basic expression in hepatocytes, and it plays a key role in lipid metabolism and glucose metabolism **(** **Figure 2C** **)**. Sortilin increases adipose mass and cholesterol absorption and decreases energy expenditure *via* the KLF4-LXR signaling pathway, leading to downregulated FGF21 and adiponectin and upregulated NPC1L1 (66). *Sort1* is also expressed on macrophages. *Sort1* deficiency could downregulate macrophage cellular cholesterol levels by reducing LDL uptake. Correspondingly, sortilin overexpression in macrophages increased the uptake of LDL and foam cell formation (14). Thus, a close correlation is observed between macrophage sortilin and lipid metabolism. Sortilin could also interact with PCSK9 to promote the secretion of PCSK9, which induces the degradation of the LDL receptor and reduces the clearance rate of LDL-C (13, 67). In addition, several studies have shown that sortilin plays an essential role in glucose metabolism. Sortilin**^−/−^** mice had enhanced glucose uptake and reduced inflammatory cytokine production compared with wild-type mice (68). Sortilin also served as a sorting partner for the glucose transporter GLUT4 to promote glucose storage (69).

## 3 ProBDNF and its receptors in MS

MS is a chronic inflammatory demyelinating disease of the CNS characterized by axonal degeneration and neurodegeneration (70) as well as an autoimmune inflammatory disease (71). The infiltration of heterogeneous cell populations such as T cells, B cells, macrophages, and microglia triggers chronic inflammatory pathological damage in the CNS (72). Based on the most extensive global study to date, a total of 2.8 million people worldwide has MS (73). MS is classified into three phenotypes: relapsing/remitting MS (RR–MS), secondary progressive MS (SP–MS) and primary progressive MS (PP–MS) (74).

Studies have shown that proBDNF induces apoptosis by activating p75^NTR^-mediated downstream signaling pathways (75). ProBDNF and mBDNF in the serum of RR–MS patients decreased, whereas truncated BDNF increased compared with healthy controls (76). Thus, low proBDNF in the serum of RR–MS patients may not be sufficient to limit the proliferation of autoreactive T cells; in addition, low mBDNF in the serum could not exert enough neuroprotection (76). Our previous study found that the expression level of proBDNF and p75^NTR^ significantly increased in the peripheral blood, spleen, and spinal cord of patients with MS and EAE model mice and co-localized with T and B cells. The administration of proBDNF-neutralizing antibodies, such as mAb-proB, can effectively improve the neurological score of EAE model mice, inhibit the expression of inflammatory cytokines in the spleen and spinal cord, reduce the percentage of T cells and B cells, and improve demyelinating lesions and inflammatory infiltration of the spinal cord. The mechanism is due to the hyperactivation of downstream NF-κB signaling by proBDNF/p75^NTR^ on peripheral human PBMCs (29). In early 2008, the New England Journal of Medicine reported that about 20.3% of patients with MS would relapse after B cell depletion therapy with anti-CD20 monoclonal antibody (77). Extensive B cell depletion therapy may lead to infusion reactions and increase the risk of serious opportunistic infections, including progressive multifocal leukoencephalopathy, bone marrow suppression, and liver damage (78). MAb-proB would not eliminate all lymphocytes in EAE model mice, indicating that mAb-proB may be a safe and effective clinical candidate drug for MS treatment.

By the end of the last century, glial cells in MS plaques have been reported to enrich for p75^NTR^ (79). Kust et al. found that the expression level of p75^NTR^ was increased in endothelial cells of the CNS of EAE model mice. The proportion of B cells, monocytes/macrophages, and segmental neutrophils was decreased in the spinal cord of p75^NTR−/−^-induced EAE. By contrast, the proportion of T cells doubled, and inflammation in the CNS was significantly enhanced. The results indicate that p75^NTR^ in endothelial cells plays a role in protecting the integrity of the blood–brain barrier and regulating immune cells in EAE model mice, particularly the interaction with T cells (80, 81). Studies have found that p75^NTR^ is expressed only on B lymphocytes (B220^+^ cells) in the brain and spinal cord of EAE model mice, whereas its expression on T lymphocytes is weak (82). P75^NTR^ does not directly act on T cells that infiltrate into the CNS of EAE model mice. In addition, Steven et al. found that p75^NTR^ is expressed on NG2-positive (an integral membrane chondroitin sulfate protein glycan expressed by oligodendrocyte progenitor cells) oligodendrocyte progenitor cells in periventricular plaques, in the subventricular zone adjacent to plaques, and in the corpus callosum of patients with MS (83). The abovementioned studies suggest that p75^NTR^ can participate in the regulation of neuroimmune pathology in EAE model mice through various pathways. In lesioned brain tissues of patients with MS and EAE mice, sortilin was highly expressed on infiltrating macrophages and activated microglia. However, the knockdown of sortilin had no effect on the progression of EAE (49). Therefore, proBDNF may play a role in the neuroimmune inflammation of MS/EAE by binding to p75^NTR^.

**Figure 3:**
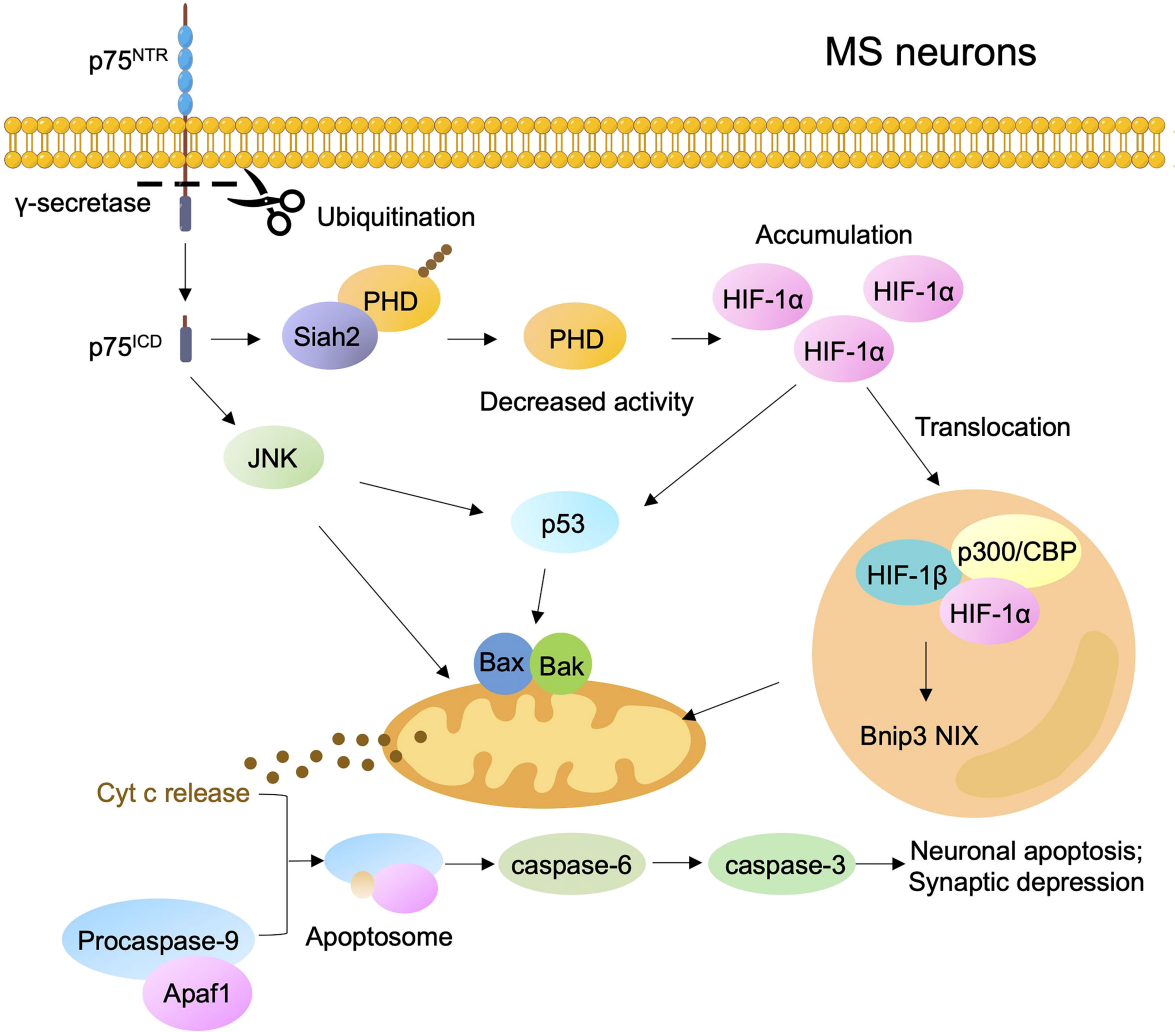
P75^NTR^ activates signaling pathways regulating neuronal death in MS during hypoxia. Hypoxia increases the γ-secretase of p75^NTR^ to release p75^ICD^ and then increases Siah2, which results in the ubiquitination and degradation of prolyl hydroxylases (PHD), thereby promoting stabilization and accumulation of HIF-1α. The pool of HIF-1α triggers mitochondrial signaling pathways to induce neuronal apoptosis by activating p53 or by up-regulating the proapoptotic gene NIX.

Several independent investigations have demonstrated that the diffused neurodegeneration in patients with MS involves mitochondrial dysfunction, such as mitochondrial respiratory chain deficiency, inadequate ATP production, and accumulated mitochondrial ROS (84–86). The accumulation of ROS and the consequent DNA and protein and lipid damage occur in hypoxia-induced neurotoxicity during MS progression. During hypoxia, p75^NTR^ undergoes oxygen-dependent cleavage by γ-secretase to provide a positive feedforward mechanism as an adaptive response to low oxygen tension (87). The cellular adaptation to hypoxia is mediated by the transcription factor hypoxia-inducible factor-1α (HIF-1α), which controls a group of genes engaging in cell migration, proliferation, metabolism, and inflammation. Hypoxia stimulates the γ-secretase-dependent release of endogenous p75^ICD^ and its interaction with absentia homolog 2 (Siah2), which decreases auto-ubiquitination. Then, Siah2 targets prolyl hydroxylases for proteasomal degradation to stabilize HIF-1α (87). The accumulation of HIF-1α triggers mitochondrial signaling pathways to induce neuronal apoptosis by activating p53 or upregulating the proapoptotic gene NIX (**Figure 3**). Therefore, targeting the oxygen-dependent cleavage of p75^NTR^ may exert potential therapeutic effects on MS. Compared with the role of proBDNF in promoting cell apoptosis by inducing cyt C release from mitochondria, BDNF contributes to endogenous neurotrophic support in MS plaques by binding to TrkB, thereby inducing the expression of NF-κB (88). Carito et al. found that polyphenolic compounds may exert neuroprotective effects and reduce the risk of MS disease by upregulating BDNF to control oxidative stress, inflammation, apoptosis, and mitochondrial dysfunction (89).

## 4 ProBDNF and its receptors in RA

RA is an autoimmune disease, wherein the immune system attacks the joints of the whole body, thereby causing joint inflammation, which, in severe cases, may lead to permanent joint damage and disability. In addition, RA may induce complications by damaging the heart, lungs, blood vessels, skin, and eyes. The prevalence of RA is approximately 0.5% worldwide, and it is higher in women (90). Our previous studies have demonstrated that mitochondria, as a key disease-related organelle, promote the pathogenesis of RA (91–93).

Recently, a study has shown that increased proBDNF and p75^NTR^ were detected in inflammatory cells of synovial tissue of patients with RA through immunohistochemistry compared with osteoarthritis. Abundant proBDNF was co-localized with p75^NTR^ in RA synovial tissue. In addition, proBDNF was co-localized with CD14^+^ monocytes and some CD20^+^ B cells in RA synovial tissue, and p75^NTR^ was primarily expressed in CD4^+^ T cells of synovial tissue. Moreover, the expression of proBDNF and its receptors, namely, p75^NTR^, and sortilin, were all higher in PBMCs in patients with RA than in healthy controls. Furthermore, the serum p75^NTR^ and sortilin levels were positively and significantly correlated with the Disease Activity Score in 28 joints (94). Intervention with p75^ECD^ can significantly decrease proinflammatory cytokines and proBDNF/p75^NTR^/sortilin in the serum and spinal cord of CIA model mice, which indicates that proBDNF/p75^NTR^/sortilin signaling promotes inflammatory response in RA (94). Our previous study found that in inflammatory pain model mice, the expression level of proBDNF and p75^NTR^ was upregulated in nerve fibers and inflammatory cells of local tissues. An anti-proBDNF antibody can relieve pain in different inflammatory pain mouse models, inhibit inflammatory cell infiltration, and activate proinflammatory cytokines (31). Therefore, we hypothesize that the proBDNF/p75^NTR^ signal derived from immune cells may be closely related to the course of RA, and inhibiting the proBDNF/p75^NTR^ signal may provide a new therapeutic strategy for improving pain in patients with RA.

**Figure 4:**
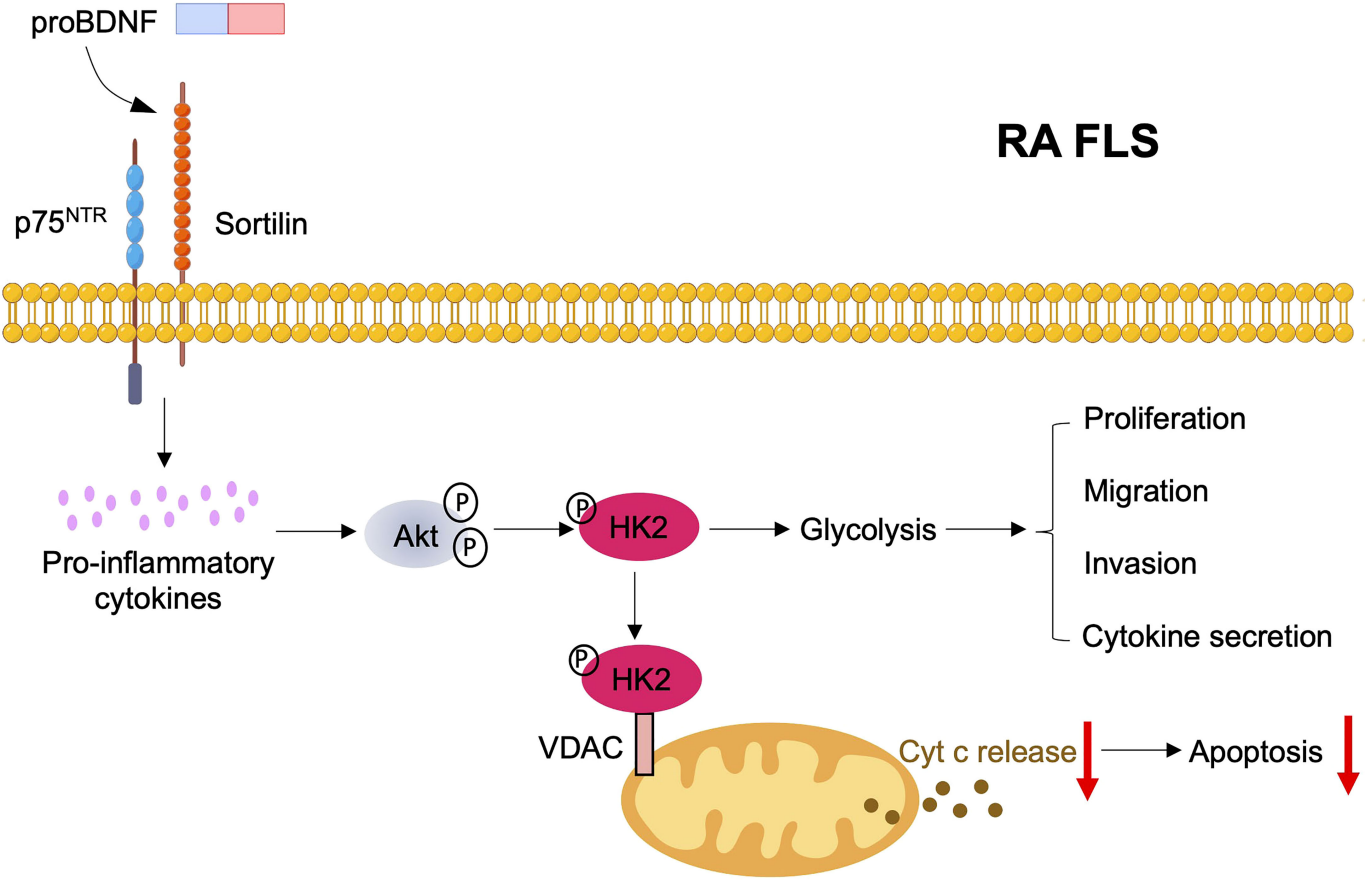
Mitochondria and glucose metabolism regulation by proBDNF/p75^NTR^/sortilin signaling in RA FLS. ProBDNF/p75^NTR^/sortilin signaling promotes the release of inflammatory cytokines, which stimulate Akt phosphorylation and then up-regulate expression and phosphorylation of hexokinase 2 (HK2). Increased binding of HK2 accompanies the phosphorylation of HK2 by Akt to mitochondrial outer membrane voltage-dependent anion channel (VDAC). Binding to VDAC enhances the affinity of hexokinases. Thus, HK2 mitochondria binding promotes glucose metabolism to induce FLS proliferation, migration, invasion and cytokine secretion, contributing to joint destruction in RA. Mitochondrial HK2 might also suppress FLS apoptosis by decreasing cytochrome C release from mitochondria.

A recent study using single-cell RNA sequencing has found that fibroblast-like synoviocyte (FLS) from active patients with RA overexpressed p75^NTR^ and sortilin compared with patients with RA in the remission stage (95). P75^NTR^ was significantly enriched in PRG4^pos^ lining FLS and THY1^pos^ COL1A1^pos^ sublining FLS, as well as remarkably expressed in FLS (95). After IL-1β stimulation *in vitro*, the expression level of p75^NTR^ on FLS of patients with RA was significantly upregulated. Consequently, p75^NTR^ signaling activated the inflammatory response in FLS, and neutralizing, or inhibiting p75^NTR^ could reduce the inflammatory factors IL-6, IL-8, and MCP1 in FLS, which was related to the activation of downstream JNK/p38 MAPK signaling (95). Chronic glucose metabolic changes induced by hypoxia and inflammatory mediators in FLS and synovial T cells will activate many signaling pathways, including MAPK, NF-κB, and PI3K/Akt pathways (96), which are crucial for the expression of adhesion molecules, secretion of cytokines, and inhibition of apoptosis, as well as for migration and invasion (96) (**Figure 4**). Therefore, proBDNF/p75^NTR^ signaling may regulate glucose metabolism, particularly in FLS and CD4^+^ T cells during RA inflammatory response. In addition, serum BDNF contributed to proinflammatory responses in patients with RA. Thus, BDNF may also play a similar role in regulating glucose metabolism and mitochondria by releasing proinflammatory cytokines (97).

In addition, sortilin is highly expressed in nine clusters of synovial macrophages, particularly in SPP1^pos^ macrophage cluster and TREM2^pos^ macrophage cluster. Based on the accumulation of mitochondrially encoded electron transport chain (ETC) subunit genes, TREM2^pos^ macrophages have stronger oxidative phosphorylation (OXPHOS) activity. On the contrary, SPP1^pos^ macrophages are more involved in the glycolytic pathway (98, 99). However, data connecting mitochondrial activity and function and sortilin in different synovial macrophage subsets must be further studied during RA pathogenesis.

## 5 ProBDNF and its receptors in SLE

SLE is an autoimmune disease of unknown etiology, which is characterized as the deposition of auto-antibodies caused by the hyperactivation of autoreactive B cells and dysregulation of antibody-secreting cells (ASCs) (100–102). In general, SLE is characterized by the inappropriate expansion of ASCs, leading to the production of auto-antibodies such as anti-DNA antibodies and antinuclear antibodies (103). ASCs can develop from multiple types of activated B cells with highly and strongly positive cell surface markers, namely, CD38 and CD27 (104–106). As ASCs develop and mature, they exhibit multiple transient cellular phenotypes to diversify their antibody repertoire and then develop distinct cell-fate endpoints (107–109). For example, ASCs with high expression of CXCR3 migrate to inflamed tissues (110). Furthermore, some subsets of ASCs such as CD27^hi^HLA-DR^hi^ (111) and TLR4^+^ CXCR4^+^ plasma cells (112) contributed to auto-antibody production and glomerulonephritis compared with other plasma cells.

Our recent study has shown that proBDNF and p75^NTR^ were upregulated in ASCs (CD19^+^ CD27^hi^ CD38^hi^). Moreover, the expression of proBDNF on ASCs has a significant clinical correlation with the auto-antibody level and disease activity of patients with SLE, indicating that ASCs^+^ proBDNF^+^ cells can be used as a clinical marker of SLE. In animal experiments, proBDNF/p75^NTR^ signaling was significantly upregulated in B cells of spontaneous and induced lupus mice. The intraperitoneal administration of mAb-proB can alleviate the condition of spontaneous and induced lupus mice, reduce the proportion of ASCs and production of auto-antibody and proinflammatory cytokines, as well as delay kidney damage (9, 10). Intraperitoneally administering pristane to induce lupus in B cell-specific p75^NTR^ knockout (CD19^cre^ p75^f/f^) mice showed that the knockout of B cell p75^NTR^ signaling can also alleviate the progression of SLE. RNA-Seq suggested the downregulation of immune-related and antibody secretion-related genes in lymph nodes of CD19^cre^ p75^f/f^ mice. R848 stimulation significantly upregulated the proBDNF/p75^NTR^ signal on B cells *in vitro*. Moreover, anti-proBDNF antibody intervention or B cell conditional knockout of p75^NTR^ could inhibit R848-induced generation of ASCs. *In vitro* intervention with mAb-proB inhibited the CpGB-stimulated B cell differentiation and production of IgG and IgM in PBMCs from healthy volunteers and patients with SLE. Therefore, the proBDNF/p75^NTR^ signaling pathway promoted the proliferation of ASCs, which plays a pathogenic role in SLE and may be a potential therapeutic biological target for SLE (9, 113). Our recent research indicated that mAb-proB inhibited the overexpansion of CD3^+^ B220^+^ cells and altered transcription levels related to cholesterol metabolism, cell cycle, and cell apoptosis, which may contribute to the attenuation of conditions of MRL/lpr lupus mice (10).

**Figure 5:**
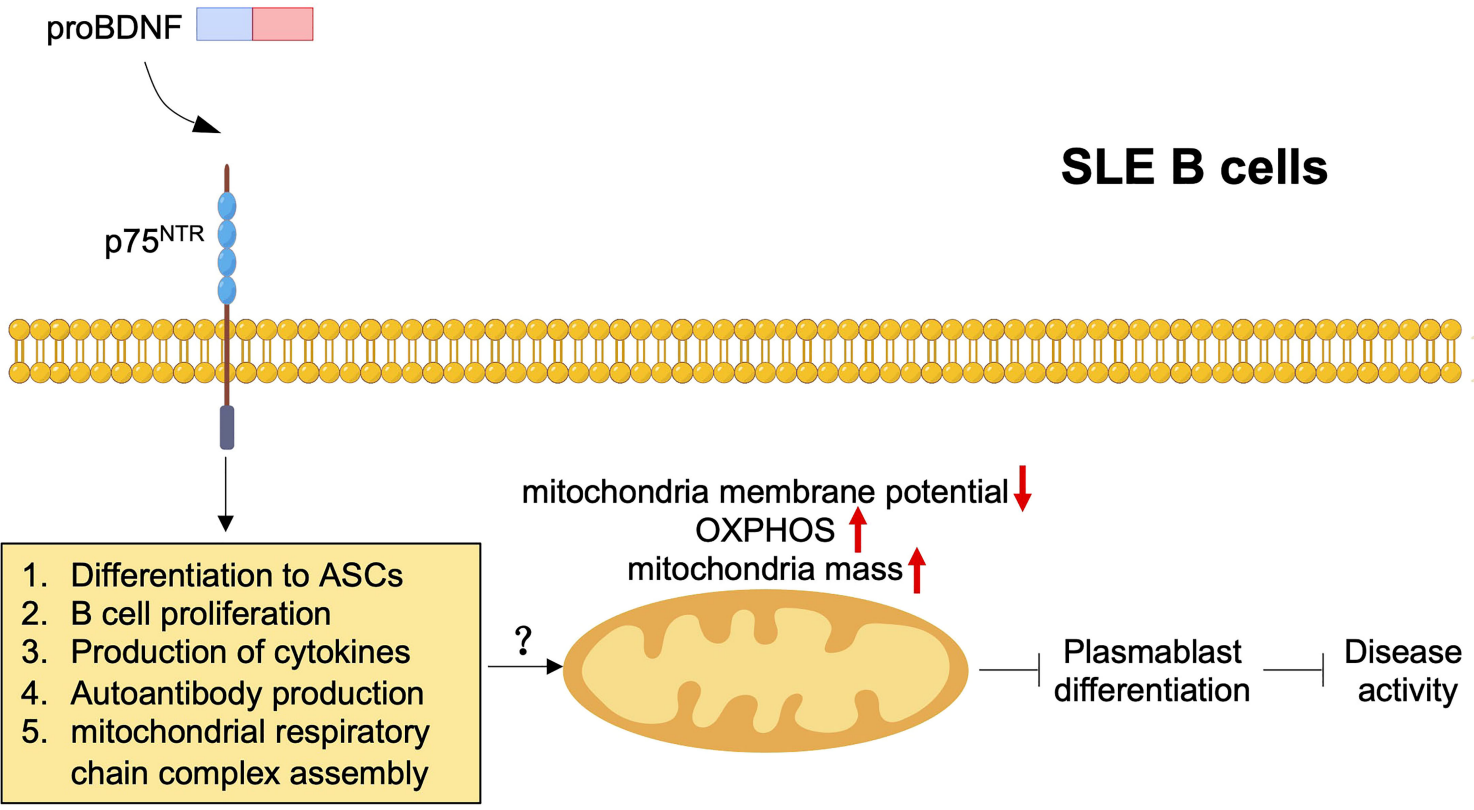
ProBDNF/p75^NTR^ signaling in SLE B cells. ProBDNF/p75^NTR^ signaling promotes B cell differentiation, proliferation, release of proinflammatory cytokines and autoantibody production and mitochondrial respiratory chain complex assembly. Mitochondria damage and dysfunction including decreased mitochondria membrane potential and enhanced OXPHOS and mitochondria mass, which may increase disease activity through inhibiting plasmablast differentiation by proBDNF/p75^NTR^ signaling pathway.

Several studies on SLE have observed mitochondrial damage and dysfunction in SLE B cells and T cells, which are characterized as enhanced mitochondrial membrane depolarization, OXPHOS, and mitochondria mass (114, 115). In addition, mitochondrial dysfunction in B cells is associated with plasmablast differentiation and disease activity in SLE (114). A previous report used spinal cord injury model mice to confirm that p75^NTR^ overexpression induced mitochondrial damage and cell apoptosis in spinal cord neurons by downregulating neurotrophic tyrosine receptor kinase 3 (116). Our study also indicated the significant difference in gene ontology of mitochondrial respiratory chain complex assembly between pristane-immunized CD19^cre^ p75^f/f^ mice and p75^f/f^ control mice by RNA-seq. Therefore, p75^NTR^ signaling may exert potential effects on mitochondrial metabolism in B cells to participate in the regulation of SLE pathogenesis (**Figure 5**). Similar to the upregulation of proBDNF, BDNF levels in B cells and serum increased in patients with SLE compared with healthy controls (117, 118). However, the level of serum BDNF and the number of BDNF^+^ B cells were independent of the SLEDAI score (117).

## 6 ProBDNF and its receptors in allergic asthma

Asthma is an immune-mediated inflammatory condition characterized by increased responsiveness to broncho-constrictive stimuli (6). Inhaled allergens can cause biphasic and reversible airflow obstruction. In the late phase of the response, activated T lymphocytes and eosinophils infiltrate in the airways because of increased bronchial reactivity (119–122). Possible sources of neurotrophins in allergic inflammation include neurons, neuron-associated cells (123–125), and immune cells, such as T cells, B cells, mast cells, macrophages, epithelial cells, and eosinophils (126–133).

Neurotrophins not only affect neurons but also interfere with functions of immune cells associated with allergies, such as mast cell degranulation, Th2 cytokine synthesis, B cell antibody production, and eosinophil survival (130, 134–136). The pathophysiological roles of neurotrophins in allergic asthma are as follows: first, elevated neurotrophin levels in bronchoalveolar fluid (BALF); second, blood neurotrophin levels correlate with airflow limitation; third, the induction of airway hyperactivity and airway obstruction through the production of ROS- and MAPK-mediated allergen-induced airway inflammation, modulation of neurite formation and cellular contractility, and proinflammatory cytokine release; finally, airway smooth muscle proliferation and matrix metalloproteinase induction (137).

Studies in animal models of allergic asthma have shown that p75^NTR^ plays a key role in the accumulation of eosinophils in the lungs. Christina et al. observed that eosinophils in peripheral blood and BALF expressed p75^NTR^ after segmental allergen provocation (138). Therefore, neurotrophins can mediate bronchial eosinophil activation by combining with p75^NTR^ and may play a role in regulating eosinophil inflammation in allergic asthma. In addition, studies showed that p75^NTR−/−^ mice had significantly reduced allergic inflammation and non-increased airway eosinophils (139, 140). Meanwhile, blocking p75^NTR^ by anti-p75^NTR^ antibody treatment can prevent eosinophilic inflammation in the lungs in mouse models (140). The activation of p75^NTR^ signaling plays a dual role in regulating the function of plasmacytoid dendritic cells (pDC), which not only reduced blood glucose levels and delayed the onset of autoimmune diabetes in RIP­CD80GP mice but also aggravated graft­*versus*­host disease in a xenotransplantation model (141). Its mechanism may be related to the activation of IRF3, IRF7, c-Jun, and IKKα/β, which reveals a novel regulatory circuit in pDC-mediated immune responses. By contrast, during allergic airway inflammation, BDNF plays a central role in modulating airway hyperresponsiveness but not the inflammatory response induced by allergen exposure (142).

## 7 ProBDNF and its receptors in T1D

Diabetes refers to a group of chronic diseases distinguished by hyperglycemia (143). The two prevalent types of diabetes are T1D and type 2 diabetes (143). The International Diabetes Federation estimates that 1 in 10 adults currently suffers from diabetes, corresponding to 537 million people worldwide. Diabetes is a major health threat, which causes 4 million deaths annually (144). Long-term hyperglycemia may lead to multiple complications, including heart diseases, nerve damage, oral health, vision loss, chronic kidney diseases, hearing loss, and impaired foot health and mental health (145). The prevalence of T1D is highest in children, although T1D can occur at any age (146). T1D is an autoimmune process that begins years before the clinical onset, with autoimmune-mediated selective damage to pancreatic β cells (147). The main clinical manifestation of T1D is hyperglycemia, which initiates polyuria, polydipsia, and weight loss. In severe cases, acute ketoacidosis may occur.

Peripheral neuropathy is a serious but often neglected complication of diabetes. The prevalence of diabetic neuropathy is as high as 50% in patients with diabetes, and it is characterized by damage of neurons, Schwann cells (SCs), and blood vessels in the peripheral nervous system (148). A study on T1D found that pancreatic sympathetic neurons contain abundant p75^NTR^ mRNA, which is directly activated on pancreatic sympathetic axons and is responsible for rapid nerve damage in patients with T1D (149). This degree of nerve damage can significantly suppress glucagon response to sympathetic activation (150, 151). Segmental axonal degeneration is secondary to p75^NTR^ activation on sympathetic axons (152), and p75^NTR^ knockout prevents most islet nerve damage (149).

The effects of oxidative stress and mitochondrial disorders in SCs on neuronal dysfunction during diabetes become more evident. In addition, long-term hyperglycemia is widely considered as a trigger of excessive ROS formation in cells, including SCs (153). Hyperglycemia increases flux through the ETC (154). Schwann cells induced by high glucose cause oxidative stress *via* intramitochondrial stress, including the overactivation of caspase-9 and Bax, and decrease Bcl-2 (155). Furthermore, a previous study utilized a mouse model of *Ngfr*-specific deletion in SCs (SC-p75^NTR^-KO) and RNA sequencing to demonstrate several metabolic pathways activated by p75^NTR^, including cholesterol metabolism and glycerolipid metabolism (148). Direct evidence has also shown that diabetic peripheral neuropathy is related to the decreased production of neurotrophins and increased p75^NTR^ expression on SCs in humans and mouse models of T1D (156–158). In an *in vitro* model of hyperglycemia stress, Tan et al. found that high glucose treatment inhibited Cav-1 transcription and protein expression within SCs, which enhanced the mitogenic response of SCs to human recombinant neuregulin-1-β1-(176–246) (NRG1-β1). NGF suppresses the glucose-induced downregulation of Cav-1 transcription and protein expression through p75^NTR^-mediated JNK activation (159). NGF/p75^NTR^ signaling increases the expression of p53 and promotes its activation by JNK in sympathetic neurons (160, 161). On the contrary, p53 could upregulate the transcription of the human *CAV-1* gene (162). Therefore, NGF/p75^NTR^ cassette modulates the response of SCs to neuregulin, which may affect the regenerative/degenerative response of these cells to hyperglycemic stress.

In the study of diabetic nephropathy (diabetic kidney disease [DKD]), Bryan et al. found that the symptoms of DKD were reduced after treating the streptozotocin-induced diabetic model mice with THX-B, a small-molecule p75^NTR^ antagonist, or a monoclonal antibody neutralizing proNGF. Diabetes increased urea and creatinine levels, decreased albumin levels in plasma, and downregulated p75^NTR^ expression in the kidney, all of which were reversed by THX-B treatment. In addition, microRNAs (miR-21-5p, miR-214-3p, and miR-342-3p) were tightly related to and highly expressed in the diabetic kidney. Moreover, the renal inflammation marker miR-146a and the elevated mRNA level of Fn-1 and Nphs, which are markers of fibrosis and inflammation, were partially, or completely reversed after THX-B or anti-proNGF mAb treatment. Therefore, p75^NTR^ antagonists and antibodies against neurotrophins may be novel tools for treating or alleviating DKD and other diabetes-associated complications (163).

BDNF also played a role in T1D. Mitsugu et al. demonstrated that the intermittent administration of BDNF ameliorated blood glucose levels in diabetic mice. The same team confirmed that BDNF reduced food intake and lowered blood glucose levels in obese diabetic animal models. Furthermore, BDNF had a hypoglycemic action independent of appetite alteration in diabetic mice. Meek et al. further confirmed that BDNF lowered blood glucose levels because of decreased glucose uptake, which is consistent with the role of p75^ICD^ but contrary to the role of sortilin in glucose uptake (164).

## 8 ProBDNF and its receptors in vasculitis

Vasculitis is a condition that covers a group of rare diseases characterized by inflammation of blood vessels, which causes organ ischemia and damage. Vasculitis usually shows a marked age tropism. For example, giant cell arteritis (GCA) predominantly affects those aged >50 years, whereas Kawasaki disease (KD) primarily affects young children (165).

Masayuki et al. found that sortilin is elevated in the acute phase but decreased in the convalescent phase of KD. The ratio of sortilin to platelet (sortilin/platelet) still increased after initial intravenous high-dose immunoglobulin treatment in unresponsive cases, whereas CRP decreased in unresponsive and responsive cases, indicating that sortilin/platelet may reflect the activity of KD more sensitively than CRP (166). In patients with GCA, the overexpression of BDNF, and their receptors was observed in the temporal artery, which may be related to the presence of proinflammatory cytokines in the inflamed arterial wall (167). Reports have demonstrated that vascular inflammation and innate immunity contribute to cardiovascular diseases such as aortic dissection (28, 168, 169). ProBDNF was upregulated in M2- but not M1-like monocytes in patients with Stanford type A acute aortic dissection (AAD). Furthermore, sera from patients with AAD promoted inflammatory responses in PBMCs from healthy controls, which was attenuated by mAb-proB treatment. Therefore, the upregulation of proBDNF in M2-like monocytes may promote the proinflammatory response in AAD (28). Furthermore, proBDNF, and BDNF, as well as its receptor, may serve as inflammatory biomarkers in vasculitis.

More but smaller mitochondria were observed in cells of the medial layer of the ascending aorta in patients with AAD compared with healthy controls, which may illustrate mitochondrial dysfunction (170). In a fluoroquinolone-induced AAD model, mitochondrial dysfunction produced more ROS and STING to promote cell apoptosis. As previously mentioned, proBDNF/p75^NTR^ signaling regulates mitochondrial function to induce cell apoptosis. Therefore, proBDNF/p75^NTR^ signaling may engage in apoptosis in the aortic wall in AAD (171).

## 9 ProBDNF and its receptors in IBD

IBD is characterized by chronic immune-mediated intestinal inflammation that is driven by genetic susceptibility as well as environmental and microbial factors, encompassing ulcerative colitis (UC) and Crohn’s disease (CD). Such diseases manifest as a relapsing–remitting course. IBD has become a global disease with increasing incidence rate worldwide in the 21^st^ century (172, 173). Some studies have shown that neurotrophins and receptors play an essential role in intestinal inflammation. Receptors for proBDNF and BDNF are common in neurons of the myenteric and submucosal plexus and mucosal endocrine cells in the gastrointestinal tract (174–176). In CD, the loss of enteric glial cells results in severe inflammation of the intestine. BDNF attenuates apoptosis of glial cells, whereas anti-BDNF antibodies significantly increase apoptosis (176). Johansson et al. (177) reported a strong correlation between massive inflammation with decreased neurotrophin immunoreaction. Furthermore, a high expression level of p75^NTR^ was observed in lamina propria cells of patients with UC (177). However, the expression and role of proBDNF in IBD remain unclear.

## 10 Conclusion

There has been a great breakthrough in therapies for IMIDs. However, new and urgent problems and difficulties in restoring immune dysregulation to normality, relieving pain more than inflammation, and exploring potential interactions between the immune and neurological system begin to exist. ProBDNF signaling promotes cell apoptosis by inducing cyt C release from mitochondria, whereas BDNF signaling exerts opposite effects by controlling oxidative stress and mitochondrial dysfunction. BDNF decreased glucose uptake in IMIDs, which is consistent with the role of p75^ICD^ but contrary to the role of sortilin in glucose uptake. Traditional studies tend to focus on the role of proBDNF and its receptors in the CNS. With the deepening of research, we have comprehensively understood the role of proBDNF and its receptors in mediating mitochondrial and metabolic pathways and in regulating the peripheral immune system in IMIDs, a large class of diseases. ProBDNF signaling usually exerts proinflammatory effects in IMIDs, whereas it exerts anti-inflammatory effects in AMI. Based on the proBDNF/p75^NTR^ signal, a series of original research was conducted, and an anti-proBDNF monoclonal mouse antibody and humanized mAb-proB with independent intellectual property rights and patents was produced. In addition, we focused on the p75^NTR^ target and prepared p75^ECD^ from prokaryotic and eukaryotic cells. In the future, we will explore the role of proBDNF and its receptors from multiple dimensions and perspectives, particularly in cellular metabolism and mitochondrial homeostasis of different immune cell subsets to further understand its mechanism involved in patients with IMID. Finally, these new insights may promote preclinical research by targeting proBDNF and its receptors in the future.

## Author contributions

QL wrote the manuscript and created the figures. ZLH revised the manuscript and figures. YZH participated in literature review and data summary. SG and PFW participated in literature review. RPD was the guarantor and revised the manuscript. All authors reviewed the final manuscript.
